# Complete genome sequence of *Bifidobacterium longum* BL020 (DSM 33815): a human stool-derived strain from the NORDBIOTIC collection

**DOI:** 10.1128/mra.00632-26

**Published:** 2026-10-12

**Authors:** Agnieszka K. Szczepankowska, Bożena Cukrowska, Tamara Aleksandrzak-Piekarczyk

**Affiliations:** 1 Institute of Biochemistry and Biophysics, Polish Academy of Sciences (IBB PAS) 90863, Warsaw, Poland; 2 Department of Research and Development, Nordic Biotic Ltd., Warsaw, Poland; Wellesley College, Wellesley, Massachusetts, USA

**Keywords:** *Bifidobacterium longum*, complete genome sequence, hybrid genome assembly, Illumina sequencing, Oxford Nanopore Technologies sequencing, probiogenomics, gut microbiota

## Abstract

We report the complete genome sequence of *Bifidobacterium longum* BL020 (DSM 33815), a human stool-derived strain from the NORDBIOTIC collection. The genome consists of a single circular chromosome of 2,413,342 bp with a G + C content of 60.0%.

## ANNOUNCEMENT

*Bifidobacterium longum* is a gram-positive anaerobe of the human gastrointestinal tract (1–4). BL020 was isolated in Japan in 1969 from the feces of a female infant. Supplier records indicate a single-colony isolate subsequently propagated in de Man, Rogosa and Sharpe (MRS) with cysteine; original isolation details are unavailable. BL020 was deposited at Leibniz Institute DSMZ–German Collection of Microorganisms and Cell Cultures (DSMZ) as DSM 33815 and included in clinically evaluated probiotic preparations (5, 6).

Lyophilized BL020 was suspended in MRS (Oxoid) with 0.05% (wt/vol) L-cysteine-HCl, plated on the same medium, and incubated anaerobically overnight at 37°C. DNA from a single-colony culture was extracted by organic extraction after lysozyme-mutanolysin and SDS-proteinase K treatment (7). Species identity was verified by Sanger sequencing of a V1–V9 16S rRNA amplicon generated with primers 27F (5′-AGRGTTYGATYMTGGCTCAG-3′) and 1492R (5′-RGYTACCTTGTTACGACTT-3′). BLASTN analysis (8) against NCBI RefSeq identified *B. longum* subsp. *longum* JCM 1217 (NC_015067.1) as the best match (100% query coverage; 99.89%–100% identity).

Illumina libraries were prepared with the NEBNext Ultra II FS DNA Library Prep Kit and sequenced on a NextSeq (2 × 150 bp), yielding 5,601,838 read pairs (11,203,676 reads; 1,680,551,400 nt). Reads were assessed using FastQC v0.11 and SeqKit v2.8.1 and trimmed/filtered using fastp v0.23.4 (9), yielding 5,403,749 read pairs. For Oxford Nanopore sequencing, DNA was sheared with a 26G needle, size-selected to remove fragments <10 kb, barcoded with the Native Barcoding Kit V14, and sequenced on a PromethION P2 Solo with an R10.4.1 flow cell. Dorado v7.6.8 generated 77,882 raw reads (N50, 11,239 nt); Porechop_ABI v0.5 (10) and Chopper v0.12.0b yielded 32,888 reads (N50, 10,059 nt), assessed using NanoPlot v1.42.

Autocycler v0.5.2 (11) combined and circularized assemblies from Flye v2.9 (12), Canu v2.3 (13), Raven v1.8.1 (14), Miniasm v0.3-r179 (15), metaMDBG v1.2 (16), NECAT v0.0.1 (17), and NextDenovo v2.5.2 (18). The assembly was polished with filtered Illumina reads using PyPOLCA v0.4.0 (19) and Polypolish v0.6.0 (20). CheckM v1.2.3 (21) estimated 100.0% completeness and 0.46% contamination. Default parameters were used unless otherwise stated. The chromosome was reoriented to *dnaA* using Dnaapler v1.3.0 (22) and evaluated using QUAST v5.0.2 (23) and Bandage v0.9 (24). The final circular chromosome is 2,413,342 bp with 60.0% G + C content. PGAP v6.10 (25) annotated 1,962 protein-coding genes, 55 tRNAs, 9 rRNAs, 3 ncRNAs, and 57 pseudogenes.

## Data Availability

The complete chromosome sequence has been deposited in GenBank under accession number JBWDMS010000001.1. The associated BioProject and BioSample accession numbers are PRJNA1437409 and SAMN56509521, respectively. Raw Illumina and Oxford Nanopore Technologies reads have been deposited in the Sequence Read Archive under accession numbers SRR37693802 and SRR37693794.
